# Errors in Sending Bills

**Published:** 1859-03

**Authors:** 


*Errors in sending Bills.*—Our kind-hearted subscribers must pardon any errors which may be made in sending them accounts, and may rest assured that they will all be satisfactorily rectified on hearing from them. In our anxiety to have the business affairs of the Journal in a proper shape, we may send bills to those who have remitted; we, however, will make no more mistakes, but some are now unavoidable. Receipts will hereafter be sent for all money remitted to the publisher.

